# The Relationship Between Foreign Language Classroom Anxiety, Enjoyment, and Expectancy-Value Motivation and Their Predictive Effects on Chinese High School Students’ Self-Rated Foreign Language Proficiency

**DOI:** 10.3389/fpsyg.2022.860603

**Published:** 2022-05-27

**Authors:** Lianqi Dong, Meihua Liu, Fan Yang

**Affiliations:** ^1^Department of Foreign Languages and Literatures, Tsinghua University, Beijing, China; ^2^Academic Affairs Office, Tsinghua University, Beijing, China

**Keywords:** foreign language classroom anxiety, foreign language enjoyment, expectancy-value motivation, self-rated foreign language proficiency, Chinese high school students

## Abstract

The present study explored the relations among foreign language (FL) classroom anxiety, enjoyment, expectancy-value motivation, and their predictive effects on Chinese high school students’ self-rated FL proficiency. Participants were 280 senior high school Chinese English as a foreign language learners who were surveyed on their foreign language classroom anxiety (FLCA), foreign language enjoyment (FLE), and expectancy-value motivation. Results showed that (1) the students generally experienced a medium to a high level of FL classroom emotions with FLE slightly higher than FLCA. They were more value-motivated than expectancy-motivated toward FL learning. Most of them perceived their FL proficiency as unsatisfying; (2) the students’ FLE was significantly positively correlated with all dimensions of expectancy-value motivation, while their FLCA and expectancy-value motivation demonstrated a complex correlation pattern. As the students’ FLCA level increased, their expectancy beliefs, intrinsic value, attainment value, and utility value decreased, but their cost value increased. By contrast, as their FLE level increased, their expectancy beliefs, intrinsic value, attainment value, utility value all increased, while their cost value first increased and then slightly decreased; and (3) expectancy beliefs, intrinsic value, private enjoyment in FL learning and anxiety arising from fear of negative evaluation jointly significantly predicted the students’ self-rated FL proficiency. Implications for future research and teaching were also discussed.

## Introduction

Foreign language (FL) learning is a demanding task requiring sustained dedication, resilience, and perseverance (Lou and Noels, 2020). Crucial to behavioral maintenance in FL learning, motivation has been long considered an indispensable contributor to FL learning success irrespective of learners’ aptitude, L1s (first languages), and FLs being learned (Dörnyei, 2008; Loh, 2019). Not surprisingly, the field of second language acquisition (SLA) is replete with motivation research paradigms, among which the expectancy-value motivation model is a less taken yet significant potential avenue for understanding L2 motivation (Nagle, 2021). Another important affective factor in FL learning success that has drawn considerable scholarly attention in recent years is FL emotion, especially propelled by the wave of positive psychology (Seligman and Csikszentmihalyi, 2000) that advocates a holistic approach to learner experience in learning. Following this has come an “affective turn” (Pavlenko, 2013; MacIntyre et al., 2019) with a growing number of FL emotions being unearthed and shot into the research limelight (see an overview by Dewaele et al., 2019a). Foreign language classroom anxiety (FLCA) and foreign language enjoyment (FLE) are the two most commonly experienced FL emotions by language learners (Dewaele and MacIntyre, 2014, 2016). Research has evidenced that whereas FLCA may have debilitating effects on FL learning, FLE can facilitate FL learning and even “undo” the adverse effects rendered by negative FL emotions (MacIntyre and Vincze, 2017). Besides being predictive of language learners’ FL proficiency, FLCA, FLE, and expectancy-value motivation correlate and interact significantly (e.g., Xu, 2017; Liu and Dong, 2021). Empirical findings concerning such an emotion-motivation relationship began to emerge recently, but the predictive effects of emotion-motivation factors remain largely under-explored. The present study aims to examine the relations among Chinese high school students’ FLCA, FLE, expectancy-value motivation, and how they predict students’ self-rated FL proficiency.

## Literature Review

### Foreign Language Classroom Anxiety

Emotions experienced by FL learners have long interested SLA researchers. Negative emotions, considered mostly debilitating in learning a FL, have first entered the research lexicon. Of the large body of FL-specific negative emotions, such as anxiety, anger, shame, disgust, hostility, and boredom, FLCA has been the most well-documented one since as early as the 1970s. There was first a Confounded Approach period regarding FLCA (MacIntyre, 2017) when inconclusive and even contradictory perceptions were common due to the lack of a specifically conceptualized FLCA paradigm. After that, following Horwitz et al.’s (1986) specification of FLCA and the introduction of the Foreign Language Classroom Anxiety Scale (FLCAS), research into FLCA has entered a Specialized Approach period. Horwitz et al. (1986) noted that FLCAS extends over three aspects: communication apprehension, test anxiety, and fear of negative evaluation. According to the authors, communication apprehension is a kind of anxiety arising from communicating with people in classrooms. Test anxiety is concerned with the worry about classroom performance associated with fear of failure. Fear of negative evaluation refers to anxious feelings about external negative evaluations, especially when exposed to evaluative situations. However, not all researchers confirmed such a three-dimension structure in their subsequent studies that instead reported a two- or four-dimension structure after factor analyses in different cultural settings (e.g., Aida, 1994; Cheng et al., 1999). Moreover, some argued that of the dimensions of FLCA, communication apprehension was the most relevant component and the strongest predictor of FL performance (Cheng et al., 1999; Shao et al., 2019), while others maintained that the fear of negative evaluation was the leading factor to trigger students’ language anxiety which could have adverse effects on language performance (He et al., 2021).

Despite such inconsistency, with FLCAS and other later developed skill-specific FLA measurements, a large volume of studies has replicated the result that FLA overall is negatively correlated with language learners’ FL learning outcomes and that FLA may negatively predict FL proficiency (e.g., Aida, 1994; Horwitz, 2001; Liu and Jackson, 2008; Zhang, 2019). In addition to this, FLA was also found to be associated with an array of learner inter/external variables in FL learning, such as learners’ age, gender, mindset, motivation, self-efficacy, learning strategy use, and teacher characteristics (e.g., Ewald, 2007; Dewaele et al., 2017, 2019b; Jiang and Dewaele, 2019; Lou and Noels, 2020). Examination of the interaction between FLA and these variables has sent the FLA research into a Dynamic Approach period (MacIntyre, 2017).

### Foreign Language Enjoyment

Toward the 21st century, since the introduction of positive psychology in the field of SLA (MacIntyre and Gregersen, 2012), a new perspective has been taken by researchers to tap into a broader range of learner emotions in FL learning. Following this, a plethora of FL emotions began to be uncovered, validated, and measured, such as enjoyment, pride, joy, hope, excitement, and boredom (e.g., Teimouri, 2018; Dewaele and Li, 2021; Li, 2021). Among them, FLE is the most researched positive emotion experienced by FL learners (Dewaele and MacIntyre, 2014, 2016). As conceptualized by Dewaele and MacIntyre (2014), FLE is the positive feeling that learners would experience in the language classroom when they are creative, overcome their limits, accomplish psychological needs, complete learning tasks and activities, have new experiences, and find themselves in a friendly instrumental environment (cited from Davari et al., 2020). To measure FLE, Dewaele and MacIntyre (2014) designed the Foreign Language Enjoyment Scale (FLES), which consists of 21 items with Likert scale ratings reflecting learners’ positive emotions toward their learning experience, peers, and teachers, establishing the research on FLE as an independent avenue in the quest to FL learner emotions. The FLES broadly measures two factors of FLE: private FLE (i.e., positive feelings about one’s own progress in FL learning) and social FLE (i.e., learners’ positive feelings about their relationships with others in FL learning; Dewaele and MacIntyre, 2016). In recent years, the FLES has undergone several rounds of revisions for clearer construct purposes (Dewaele and MacIntyre, 2016) and validations, and has been applied in varying FL learning contexts, such as in Germany (Resnik and Dewaele, 2021), Iran (Elahi Shirvan and Taherian, 2021), and China (Li et al., 2018; Jiang and Dewaele, 2019; Dewaele and Li, 2022). The two broad components of FLES have been retained across studies, although researchers further differentiated FLE-Teacher and FLE-Atmosphere (Li et al., 2018), or FLE of Teacher Support and FLE of Student Support (Jin and Zhang, 2018) within the dimension of social FLE. Particularly, research has found that private FLE was significantly correlated with language learners’ amount of FL use both inside and outside the classroom and that the effect of private FLE was stronger and more straightforward than that of social FLE (Saito et al., 2018).

Unlike FLCA, FLE is positively associated with FL learners’ learning outcomes (Dewaele and MacIntyre, 2014; Dewaele and Alfawzan, 2018; Jiang and Dewaele, 2019). What’s more, it is posited that FLE is the emotional key to unlocking learners’ potential and could help sustain an enjoyable and safe psychological atmosphere especially when learners are in the face of unfamiliar languages and cultures (Dewaele and MacIntyre, 2014). As with FLCA, FLE also interrelates to a wide display of FL learner variables, such as learners’ gender, age, multilingualism, and FL teacher variables, including teacher’s age, gender, accent, and strictness (Dewaele et al., 2017, 2019b).

Additionally, FLCA and FLE are found to be independent emotional dimensions bearing a “right-and-left feet” relationship in FL learning (Dewaele and MacIntyre, 2016; Boudreau et al., 2018). In their study, Li et al. (2020) found three different interaction patterns between the two continuously fluctuating emotions. Research has also shown that FLCA over time may gradually evolve into a stable learner trait and hence is not easily malleable while FLE appears to be more sensitive to factors from learners’ FL learning environment, such as teachers’ behaviors, stimulus from peers, and FL instruction style (Dewaele et al., 2017; Saito et al., 2018). Therefore, it is argued that FLE may cluster to exert cumulative and positive effects on FL learning in the long run (Saito et al., 2018). Despite this, however, it is not that positive emotions will do all wonders for FL learning. Rather, it may be more reasonable that FL learners maintain a proper positive-to-negative ratio (also positivity ratio) or an emotional balance (Dewaele and MacIntyre, 2014), just as we need to walk with both feet.

### Expectancy-Value Motivation

Learner motives are fundamental to the acquisition of an additional language. Foreign language motivation reflects FL learners’ driving force toward and perseverance with FL learning (Gardner and Lambert, 1972; Gardner, 1985). Over the past 60 years, the research on FL learning motivation has been vibrant. As a result, a broad spectrum of L2 motivation paradigms sprung up one after another, principally represented by the two peaks of Gardner’s (1985) socio-psychological/educational motivation model and Dörnyei’s (2008) L2 Motivational Self-System (L2MSS; Boo et al., 2015).

In recent years, both Gardner’s L2 motivation model and Dörnyei’s L2MSS have been under some criticism (see Oga-Baldwin et al., 2019; Nagle, 2021), chiefly due to the former’s over-emphasis on integrative-ness and the latter’s lack of predictive power on FL learning proficiency. Oga-Baldwin et al. (2019) reviewed major SLA motivation models, arguing for the need to consider learners’ competence beliefs when researching L2 motivation, an important element absent from the work of most L2 theorists. Taking into account learners’ competence beliefs (or the expectancy component of motivation, the two terms will be used interchangeably in the present study), expectancy-value theory (EVT) is a long-standing perspective on motivation in the field of educational psychology initially pioneered by Atkinson (1957) and further developed by Wigfield and Eccles (2000) and Eccles and Wigfield (2020). According to the EVT, individuals’ motivation to do a task is the product of two key factors. One is their expectancy of success, and the other is their value of success in the task. The two components function in a multiplicative (1 + 1 > 2) fashion to jointly predict learners’ choices, effort, persistence, and performance in learning tasks (Wigfield and Eccles, 2000).

As defined in Eccles and Wigfield (2020), expectancy for success concerns individuals’ competence beliefs about how well they have done and will do on an upcoming task about a specific learning activity. Both cross-sectional and longitudinal studies revealed significant positive correlations between learners’ expectancy beliefs and learning performance/proficiency (e.g., see Loh, 2019). Unlike expectancy beliefs, subjective task values (encompassing Attainment Value, Utility Value, Cost Value, and Intrinsic Value) mainly pertain to individuals’ rationale for choosing and persevering with a specific learning activity (Wigfield and Eccles, 2000).

Of the four types of aforementioned value beliefs, attainment value is concerned with to what extent individuals perceive the importance of doing well in a task. Wigfield and Eccles (2000) noted that attainment value is related to one’s personal (mastery and performance) goals. Utility value is individuals’ evaluation of the usefulness of a task regarding their current or future goals (Eccles and Wigfield, 2020). Intrinsic value reflects one’s inner gains from engaging in and completing a task. Empirical research has indicated that attainment value, utility value, and intrinsic value are all significant positive predictors of learning success (Loh, 2019). Cost value, however, reflects individuals’ evaluation of how much effort, opportunity, and emotional cost will be required if they are to complete a task. Individuals tend to avoid tasks that cost too much relative to their benefits and a high cost value appraisal may lead to giving up if it exceeds one’s ability beliefs (Loh, 2019; Eccles and Wigfield, 2020).

These four separate facets of subjective task value combine to predict an individual’s learning engagement and effort. As such, the expectancy and value components of motivation each have a distinct yet interconnected role in determining learners’ overall learning motives (Nagle, 2021). Evidence from educational psychology has explicitly demonstrated that expectancy of success significantly predicted adademic achievement, with value appraisals more predictive of motivated behaviors and persistence (e.g., Guo et al., 2017). In the field of SLA, though the application of EVT in L2 motivation research has remained a less-traveled path, extant research has already provided some illuminating findings. For instance, Nagle (2021) examined links between dimensions of expectancy-value motivation and university students’ effort, persistence, and achievement in a Spanish course. The author found that the participants’ willingness to communicate in learning the Spanish course was predicted by their attainment value and intrinsic value, that the likelihood of continued course enrollment was predicted by their intrinsic value, and that the course achievement was predicted by their expectancy beliefs. Meanwhile, reseachers also noted that competency beliefs and intrinsic value are two synchronous ideas for many young students and that expectancy belief has a stronger correlation with intrinsic and attainment value but a weaker association with both utility and cost value (Loh, 2019). Recently, empirical findings supported the existence of the “expectancy × value” interaction in the context of FL learning, adding that such interaction is not only value-specific (Wan, 2019) but also activity-specific (Zhan et al., 2020), which implies that the patterns of “expectancy × value” interaction in FL learning can vary across tasks, activities, and contexts. Nevertheless, still more empirical evidence is needed to further verify this argument (Eccles and Wigfield, 2020).

### Foreign Language Classroom Anxiety, Foreign Language Enjoyment, and Expectancy-Value Motivation

Given the relevance of FL emotions and expectancy-value motivation to FL learning, researchers showed interest in finding out how FLCA, FLE, and expectancy-value motivation are linked. Some researchers analyzed correlations between FL emotions and components of expectancy-value motivation. For example, Xu (2017) found that Chinese first-year undergraduates’ FLA was negatively correlated with their expectancy and intrinsic value but positively connected with attainment value. Other researchers (e.g., Liu and Dong, 2021; Zhang, 2021) further revealed that expectancy-value motivation could predict FL learning emotions. The longitudinal study by Liu and Dong (2021) revealed that both expectancy and value components of motivation were negative predictors for anxiety experienced by postgraduates learning academic oral English. These prior research findings inform that it may be more discreet to consider the relations between FL emotions and expectancy-value motivation as bidirectional since each can play a role in affecting the other, and they both are critical to FL learning success.

### Foreign Language Classroom Anxiety, Foreign Language Enjoyment, Expectancy-Value Motivation, and Foreign Language Proficiency

With the links between FL emotions and motivation beginning to draw scholarly attention recently, inquiries were extended to understand how FL emotions and expectancy-value motivation could simultaneously predict FL learning. Xu’s (2017) study with first-year Chinese undergraduates showed that FLA, expectancy beliefs, and intrinsic value could jointly predict FL listening test scores, but the research did not tap into the effects of the “expectancy × value” interaction. In a recent study, Dong and Liu (2022) examined the effects of FLE on FL test performance among Chinese high school students and found that the expectancy component of motivation mediated the predictive effects of FLE and that the “expectancy × value” interaction existed in the pathway. With a holistic view of learners’ FL emotions gaining momentum, we currently know little about how expectancy-value motivation may predict FL learning outcomes when diverse FL emotions are considered synchronously, which motivated the present study.

## The Present Study

Although the significance of FL emotions, FL expectancy-value motivation, and the links between them have been acknowledged in the literature, the discussion can be advanced in some more respects. First, a holistic view looking into the two emotional feet in FL learning, namely both FLCA and FLE and examining their relations with FL expectancy-value motivation and combined predictive effects on FL learning is evidently lacking. This is worthy of our attention because, for one thing, in a real FL learning context, FL learners do not experience one type of emotion at a single time but are in the constant flow of different emotions; and for another, the two FL emotions intertwine significantly with motivation along the FL learning process (Liu and Dong, 2021). Second, although EVT holds significant potential for understanding L2 motivation (Nagle, 2021), SLA research that takes such a perspective is scarce. Moreover, though it was conceptualized that learners’ expectancy for success and subjective task values multiplicatively predict learning performance and proficiency, such “expectancy × value” interaction has not been sufficiently validated in different contexts, and their effects on FL learning and proficiency remain a less-understood myth. Third, the past research has primarily been conducted among FL learners at a tertiary education level (e.g., Liu and Dong, 2021; Zhang, 2021), leaving FL emotions and expectancy-value motivation of young FL learners largely unnoticed, although young FL learners’ FLCA, FLE, and expectancy-value motivation appraisals can be very different from college students (Li et al., 2018; Loh, 2019).

Currently, the number of Chinese high school students learning a FL as a compulsory course has reached more than 41 million (China Ministry of Education, 2021). Given the lack of research mentioned above, it is crucial to establish links between FLCA, FLE, and expectancy-value motivation, and investigate their joint predictive effects on Chinese young learners’ FL learning. Focusing on FL learners of this population, the present study aims to seek answers to the following research questions (RQs):

RQ1. What is the general profile of Chinese high school students’ FLCA, FLE, expectancy-value motivation, and self-rated FL proficiency?
RQ2. How are Chinese high school students’ FLCA and FLE related to their expectancy-value motivation?
RQ3. How do FLCA, FLE, and expectancy-value motivation predict Chinese high school students’ self-rated FL proficiency?

## Materials and Methods

### Participants

The study focused on young FL learners who were 280 (148 male and 132 female) English as a foreign language (EFL) learning students from a senior high school in Northwestern China, ranging from 15 to 20 years old (*M* = 17.6 years; SD = 0.93). They were all native Chinese speakers. Aiming to get high scores in English, all participants needed to take frequent tests on English, monthly or bimonthly. High scores on tests are deemed to be reflective of their English learning success. Before the assessment session, the participants were familiarized with the purpose of the study. They were apprised of the nature of voluntary participation, with consents obtained from them, their headteachers, and the school.

### Measures

#### Background Information Questionnaire

A 3-item background information questionnaire was designed to collect the participants’ personal information, including age, gender, and class number.

### English Learning Expectancy-Value Motivation Scale

The English Learning Expectancy-Value Motivation Scale was an 18-item measure adapted from the Expectancy-Value Beliefs Inventory (EVBI; Trautwein et al., 2012). The original EVBI consisted of 16 items covering five dimensions: expectancy, intrinsic value, attainment value, utility value, and cost value. Example items include “I would like to take more English classes” and “I always look forward to English classes.” To fit the present situation, we added the item “Good grades in English can be of great value to me later” to the dimension of utility value and “The amount of time I spend on learning English keeps me from doing other things I would like to do” to the cost value dimension according to the recent modifications in Yang and Mindrila (2020). For this reason, both confirmatory factor analysis (CFA) and exploratory structural equation modeling (ESEM) were conducted to examine the factor structure of the newly formulated measurement as recommended by Alamer (2022) and Alamer and Marsh (2022). All items were placed on a 7-point Likert scale, ranging from “Strongly Disagree” to “Strongly Agree.” The higher the score, the stronger the expectancy beliefs and subjective values. Reliability analysis revealed high internal consistency of the scale in this study (Cronbach alpha = 0.89).

### Foreign Language Classroom Anxiety Scale

The study adopted the English Language Classroom Anxiety Scale, an 8-item measure extracted from the FLCAS (Horwitz et al., 1986) by Jiang and Dewaele (2019) in their research on Chinese EFL learners’ FLCA. As Jiang and Dewaele (2019) maintained, this reduced version reflected physical symptoms of anxiety, nervousness, and lack of confidence rendered by FLCA, thus it suits the purpose of the present study which aims to understand young FL learners’ anxiety in the classroom learning. The authors did not report the dimensions of the measure. For this reason, factor analysis was performed in the present study. All the items were placed on a 7-point Likert scale, and the higher the score, the higher levels of the participants’ FLCA. Example items included “I always think that the other students learn English better than I do” and “Even if I am well prepared for the English class, I feel anxious about it.” The scale also exhibited high internal consistency in this study (Cronbach alpha = 0.80).

### Foreign Language Enjoyment Scale

The English Language Enjoyment Scale, a 10-item measure extracted from the FLE (Dewaele and MacIntyre, 2014) by Jiang and Dewaele (2019) in their quest to investigate Chinese college EFL learners’ FLE, was adapted and used in this study. The reasons are twofold. First, as Jiang and Dewaele (2019) commented, these items reflected both social and private dimensions of FLE in classroom learning, which is in line with the context of the present study. Second, for an in-depth discussion, we seek to compare Chinese high school students’ FL classroom emotions with those of Chinese college students. All items were positively phrased on a 7-point Likert scale, and a higher score indicates a higher level of FLE. Example items included “I performed well in this term’s English class.” and “My English class is a positive learning environment.” The scale’s internal consistency in this study was also high (Cronbach alpha = 0.86).

### Self-Rated Foreign Language Proficiency

Participants self-rated their current English proficiency (score range: 1–10) in the questionnaire session. The question for eliciting their response was “If the full score is 10, how much would you rate your current English proficiency?”

### Data Collection

The study was conducted in the fourth week of the semester when the participants might form appraisals for themselves and have adapted to the EFL courses they were taking. The language of the questionnaire was Chinese, and the translation was double-checked. The participants completed the composite questionnaire and self-rated their current English proficiency. The session lasted for about 20 minutes with the help of two teachers from the school.

### Data Analysis

The survey data were mainly analyzed using SPSS 26.0 and Mplus 8.3 which examined the reliability and validity of measures, the central tendency of data, correlations, and predictive effects of variables. After the main data analysis, we used Microsoft Excel 16 to draw figures that reflected the associations among FLCA, FLE, and expectancy-value motivation.

### Factor Structure of the Foreign Language Classroom Anxiety Scale and Foreign Language Enjoyment Scale

A multivariate normal distribution test was performed before we dealt with all the standardized data. The results of the Shapiro–Wilk test (*W* = 0.989, *p* > 0.05) and skewness and kurtosis value altogether indicated that the data showed a normal distribution. We began by examining the factor structures of the FLCAS and FLES. The results of Kaiser-Meyer-Olkin (KMO) tests for the FLCAS and FLES were 0.80 and 0.85, respectively, suggesting that both scales were suitable for factor analysis. A rotated factor analysis (varimax) on the English Language Classroom Anxiety Scale generated two factors: Communication Anxiety (FLCAS1, 37.22% variance) and Fear of Negative Evaluation (FLCAS2, 18.75% variance), explaining a total of about 55.97% of participants’ FLCA variance. Principle component analysis on the English Language Enjoyment Scale reported two factors: FLES-social (FLES1, 34.09% variance) and FLES-private (FLES2, 26.45% variance), explaining a total of about 60.54% of FLE variance. To assess the construct validity of the FLCAS and FLES, we employed the ESEM method which allowed items to be freely estimated and cross-loaded, and was considered an improved method integrating the merits of both CFA and EFA (exploratory factor analysis; see Alamer, 2021b,^2022^). The results confirmed the two dimensions of the FLCAS and the FLES with model fit indices for both scales at an acceptable level (Alamer and Marsh, 2022).

### Factor Structure of the Expectancy-Value Motivation Scale

As previously mentioned, the original English Learning Expectancy-Value Motivation Scale was adapted and two extra items from Yang and Mindrila (2020) were incorporated. Thus, we confirmed the factor structure of the new measurement and assessed its construct validity (see Table 1).

**TABLE 1:** CFA and ESEM model fit indices for the expectancy-value motivation scale.

Model	χ^2^	*p*	*df*	SRMR	RMSEA	CFI	TLI
CFA	266.69	<0.001	112	0.07	0.07	0.91	0.93
ESEM	125.19	<0.001	73	0.02	0.05	0.98	0.95

The results in Table 1 show that although both CFA and ESEM models generated acceptable model fit indices (i.e., CFI > 0.90, TLI > 0.90, SRMR ≤ 0.07, and RMSEA ≤ 0.07), the ESEM solution is more desirable and differences between fit values are above the typical criterion (i.e., differences in CFI > 0.015, see Alamer and Marsh, 2022). Therefore, the ESEM framework of expectancy-value motivation was used in the present study, and its standardized factor loadings are presented in Table 2.

**TABLE 2:** ESEM factor loadings of the expectancy-value motivation scale.

Items	Attainment	Intrinsic	Utility	Cost	Expectancy
Attainment 1	0.45*	0.33	0.00	0.19	–0.02
Attainment 2	0.87*	0.02	0.05	0.02	0.00
Attainment 3	0.65*	–0.02	0.08	0.22	0.01
Intrinsic 1	0.26	0.69*	–0.04	–0.12	0.05
Intrinsic 2	0.00	0.82*	–0.04	–0.03	–0.03
Intrinsic 3	–0.08	0.76*	0.13	0.05	0.05
Intrinsic 4	–0.13	0.88*	0.06	0.10	0.01
Intrinsic 5	0.15	0.68*	0.06	0.05	–0.01
Intrinsic 6	0.09	0.48*	–0.24	–0.06	0.25
Cost 1	0.22	0.25	–0.06	0.57*	0.04
Cost 2	0.03	0.38	–0.01	0.57*	–0.06
Cost 3	0.01	–0.03	0.32	0.67*	0.03
Utility 1	0.02	0.01	0.83*	–0.04	0.05
Utility 2	0.03	–0.01	0.82*	0.05	–0.08
Utility 3	0.03	0.19	0.63*	–0.08	–0.02
Expectancy 1	0.01	0.01	–0.07	–0.19	0.67*
Expectancy 2	–0.02	–0.03	0.00	0.02	0.95*
Expectancy 3	0.01	0.10	0.05	0.11	0.76*

*p < 0.05.

## Results

### Means, SDs, and Correlations of Foreign Language Classroom Anxiety, Foreign Language Enjoyment, Expectancy-Value Motivation, and Self-Rated Foreign Language Proficiency

Table 3 reports descriptive and Pearson correlation statistics of the main variables. As shown, the mean of the participants’ self-rated FL proficiency was relatively low (M = 4.73) on a 1–10 scale, which means that the participants generally perceived their FL proficiency as unsatisfying. Their experience of classroom emotions was at a medium to a high level over the mid-point of 3.5 (7-point Likert scale), with the FLE slightly higher than the FLCA. Compared to their value component of motivation, the participants’ expectancy beliefs were significantly weaker, indicating that they were more motivated by the value of learning the FL. Of their four value components of motivation, the utility value was the highest, and the intrinsic value was the lowest, suggesting that the participants were more instrumentally motivated than intrinsically motivated toward their FL learning.

**TABLE 3:** Summary of means, standard deviation, and correlations among main variables.

Variables	Mean	SD	1	2	3	4	5	6	7	8	9
1. Self-ratings	4.73	1.55	1								
2. FLCAS-neg	4.38	1.27	−0.40**	1							
3. FLCAS-com	4.48	1.17	−0.26**	0.37**	1						
4. FLES-social	4.68	1.10	0.26**	−0.18**	0.05	1					
5. FLES-private	4.57	1.03	0.58**	−0.38**	−0.18**	0.55**	1				
6. Expectancy	4.00	1.50	0.54**	−0.26**	−0.46**	0.14**	0.40**	1			
7. Intrinsic	4.13	1.21	0.67**	−0.40**	−0.24**	0.44**	0.72**	0.56**	1		
8. Attainment	5.38	1.14	0.35**	−0.22**	0.04	0.39**	0.50**	0.23**	0.53**	1	
9. Cost	5.03	1.23	–0.02	0.04	0.33**	0.24**	0.19**	-0.21**	0.15*	0.35**	1
10. Utility	5.67	1.14	0.25**	−0.16*	0.01	0.36**	0.49**	0.19**	0.48**	0.66**	0.35**

*Note: FLCAS-neg, foreign language classroom anxiety arising from fear of negative evaluation; FLCAS-com, foreign language classroom communication anxiety.*

**Correlation is significant at the 0.01 level (2-tailed). *Correlation is significant at the 0.05 level.

In addition, the participants’ self-rated FL proficiency was significantly positively correlated with FLE and almost all dimensions of expectancy-value motivation (except for the cost value) and significantly negatively correlated with FLCA (*p* < 0.01). Dimensions of FLE and FLCA were significantly positively intra-correlated while significantly negatively inter-correlated, except for the correlation between FLE-social and communication anxiety being not significant. The participants’ FLCA was significantly negatively correlated, while FLE was positively correlated with their expectancy beliefs. Specifically, the correlation between FLCA arising from fear of negative evaluation and expectancy beliefs showed a small to medium effect size (*r* = -0.26), and that between FL classroom communication anxiety and expectancy beliefs exhibited a medium to large effect size (*r* = -0.46) (Plonsky and Oswald, 2014). With a unitary pattern, dimensions of FLE were significantly positively correlated with all factors of expectancy-value motivation. The correlation between intrinsic value and private FLE showed a large effect size (*r* = 0.72) while that between cost value and the two dimensions of FLE only reported small effect sizes (*r* = 0.19 ∼ 0.24). The correlational relations between the dimensions of FLCA and expectancy-value motivation were rather complex: intrinsic value was negatively correlated while cost value was positively correlated with the dimensions of FLCA, and attainment value and utility value both were negatively correlated with FLCA caused by fear of negative evaluation (small effect sizes, *r* = -0.16 ∼ -0.22) but positively correlated with FLCA arising from communication (small effect sizes, *r* = 0.01 ∼ 0.04).

### Relationship Between Foreign Language Classroom Anxiety, Foreign Language Enjoyment, and Expectancy-Value Motivation

**FIGURE 1:**
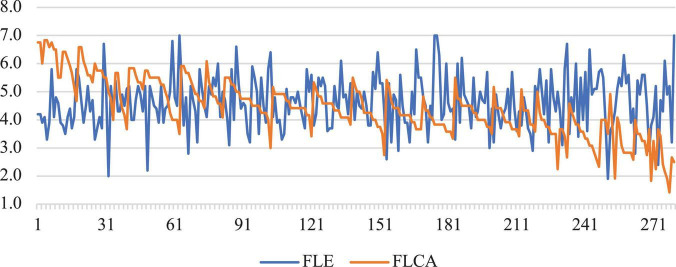
Relationship between FLCA and FLE across individuals.

After a bird’s view of the links between FLCA, FLE, and expectancy-value motivation components, a more detailed analysis was performed to probe more relational sophistication among these variables. Figure 1 profiles the links between individual participants’ FLCA and FLE (numbers on the horizontal axis refer to participants’ ID and numbers on the vertical axis refer to the strength of FL emotions). As displayed, these two FL emotion feet were stably and negatively associated across individuals and the pattern curves of them are not asymmetric. As such, FLCA and FLE were indeed independent dimensions that evolved within related but distinct systems. Meantime, individual differences were also remarkable in the experience of these two FL emotions.

**FIGURE 2:**
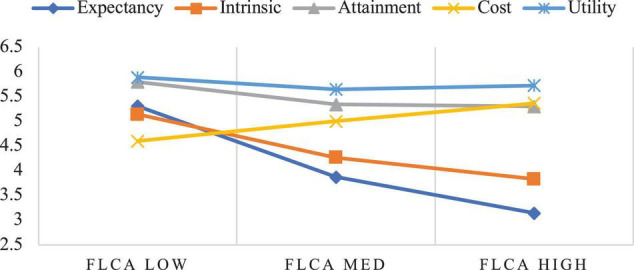
Relationship between FLCA and expectancy-value motivation.

Figure 2 presents how the participants’ components of expectancy-value motivation were linked with their FLCA. As can be seen, irrespective of their levels of FLCA, the participants generally held relatively high utility value appraisals in FL learning. More precisely, the participants of high FLCA had an almost equally high cost and attainment value, followed by intrinsic value and expectancy beliefs; the participants of medium FLCA held high attainment value, followed by cost value, intrinsic value, and expectancy beliefs; and the participants of low FLCA had high attainment value, followed by expectancy beliefs, intrinsic value, and cost value. Overall, there appeared a pattern that as the participants’ level of FLCA increased, their expectancy beliefs, intrinsic value, attainment value, and utility value all decreased, while their cost value increased. Moreover, the changes in participants’ FLCA were conspicuously accompanied by the changes in expectancy beliefs, intrinsic value, and cost value.

**FIGURE 3:**
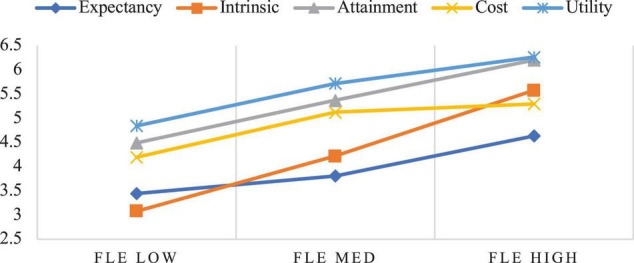
Relationship between FLE and expectancy-value motivation.

Figure 3 depicts the relation between the participants’ FLE and components of expectancy-value motivation. Similarly, the participants’ levels of expectancy-value motivation varied by their levels of FLE. Despite the FLE differences, the participants had relatively high utility value and attainment value in FL learning, which is almost the same as when the participants’ FLCA was examined. The participants with high FLE held high intrinsic value, followed by cost value and expectancy beliefs; the participants with medium FLE had high cost value appraisals followed by intrinsic value and expectancy beliefs; and the participants with low FLE held high cost value appraisals followed by expectancy beliefs and intrinsic value. Further, it can be observed that as participants’ level of FLE increased, their expectancy beliefs, intrinsic value, attainment value, and utility value all increased while their cost value first increased and then slightly decreased. Differentiating from Figure 2, the changes in the participants’ FLE were markedly echoed by the changes in all components of expectancy-value motivation.

### Predictive Effects of Foreign Language Classroom Anxiety, Foreign Language Enjoyment, and Expectancy-Value Motivation on Self-Rated Foreign Language Proficiency

A stepwise hierarchical multiple regression analysis was performed to test the predictive effects of the participants’ FLCA, FLE, and expectancy-value motivation on their self-rated FL proficiency. The averages of the four FL classroom emotion factor scores and the five expectancy-value factor scores were used as predictor variables, and the average of the self-rated FL proficiency scores was used as the dependent variable. The analysis generated four statistically significant models. Model 2 with intrinsic value and expectancy beliefs as predictor variables (Adjusted *R*^2^ = 0.49) accounts for approximately 49% of the total self-rated FL proficiency variance, significantly more than Model 1 with intrinsic value as a single predictor (Adjusted *R*^2^ = 0.45). Model 3 with intrinsic value, expectancy beliefs, and FLCAS-neg as predictor variables (Adjusted *R*^2^ = 0.51) accounts for approximately 51% of the total variance, significantly more than Model 2 (Adjusted *R*^2^ = 0.49). Model 4 with intrinsic value, expectancy beliefs, FLCAS-neg, and FLES-private as predictor variables (Adjusted *R*^2^ = 0.52) accounts for approximately 52% of the total variance, significantly more than Model 3 (Adjusted *R*^2^ = 0.51). As the VIF indexes indicated, no significant collinearity among variables was detected. Of the four predictors in Model 4, the participants’ expectancy beliefs, intrinsic value, and FLE-private were strong positive predictors while FLCAS-neg was a negative predictor of their self-rated FL proficiency.

Drawing on Zhan et al. (2020) and following the “expectancy × value” interaction reviewed in the literature, we further examined whether “expectancy × value” interaction existed with FLCA and FLE in predicting self-rated FL proficiency. To this end, we generated the “expectancy × intrinsic value” variable, added it to the existing predictor variables, and conducted another round of stepwise hierarchical multiple regression analysis. This time, four models were yielded. Yet the “expectancy × intrinsic value” variable didn’t enter any regression model and the *R*^2^ changes of new models were not significant compared with those in Table 4. This being said, the “expectancy × value” interaction did not appear to predict the participants’ self-rated FL proficiency.

**TABLE 4:** Regression models with self-rated FL proficiency as a dependent variable.

		Unstandardized coefficients		Standardized coefficients				
		*B*	Std. Error	Beta	*t*-value	*p*-value	VIF	Adjusted *R^2^*
Model 1	(Constant)	1.08	0.25		4.36	0.00		0.45
	Intrinsic value	0.87	0.06	0.67	15.23	0.00	1.00	
Model 2	(Constant)	0.79	0.25		3.21	0.00		0.49
	Intrinsic value	0.70	0.07	0.54	10.45	0.00	1.46	
	Expectancy	0.25	0.05	0.24	4.67	0.00	1.46	
Model 3	(Constant)	1.93	0.42		4.55	0.00		0.51
	Intrinsic value	0.63	0.07	0.48	9.05	0.00	1.62	
	Expectancy	0.25	0.05	0.23	4.60	0.00	1.46	
	FLCAS-neg	-0.19	0.06	–0.15	–3.28	0.00	1.19	
Model 4	(Constant)	0.28	0.48		2.68	0.01		0.52
	Intrinsic value	0.48	0.09	0.37	5.47	0.00	2.62	
	Expectancy	0.25	0.05	0.24	4.71	0.00	1.46	
	FLCAS-neg	-0.16	0.06	–0.13	–2.86	0.01	1.22	
	FLES-private	0.26	0.09	0.17	2.82	0.01	2.13	

*Note: FLCAS-neg, foreign language classroom anxiety arising from fear of negative evaluation.*

## Discussion

### General Profile of Chinese High School Students’ Foreign Language Classroom Anxiety, Foreign Language Enjoyment, Expectancy-Value Motivation, and Self-Rated Foreign Language Proficiency

RQ1 focuses on the general profile of Chinese high school students’ FLCA, FLE, expectancy-value motivation, and self-rated FL proficiency. The results showed that the participants, in general, experienced a medium to a high degree of FL classroom emotions and that their FLE was slightly higher than FLCA, which is consistent with most previous findings (e.g., Dewaele and MacIntyre, 2014; Jiang and Dewaele, 2019; Dewaele and Li, 2022). However, compared with the levels of FLCA and FLE reported by Chinese college students in Jiang and Dewaele (2019), the level of FLCA was higher while that of FLE was lower among high school students in the present study. This might be because college students in China often have a relatively higher degree of autonomy over their FL learning. In contrast, high school students usually have to follow a prescribed learning process and face greater challenges in a more exam-oriented environment (Yao et al., 2021). For instance, the participants of the present study had to take at least four large-scale English examinations in a single semester that aim to assess their mastery of English grammar, reading, writing, and other knowledge and/or skills required by the national curriculum. Consequently, they were more anxious and less enjoyable in FL learning.

Additionally, it was found that the participants were more value-motivated, especially driven by utility and attainment value, but less expectancy-motivated in FL learning, which also echoes some previous research (e.g., Liu, 2007; Gan, 2009). This implied that most Chinese high school students perceived FL learning more as helpful and important to their future goals even if they are not interested in it. This phenomenon, in effect, is common among Asian students (Loh, 2019) since most of them work hard on schoolwork mainly because they want to please their parents and conform to certain social norms, such as meeting the expectations of others and getting admissions to top universities. Still, the participants perceived FL learning as a challenging task and regarded their FL proficiency as unsatisfying, which was even common among most Chinese college students (e.g., Zhan et al., 2020). Although the participants may likely underestimate their FL proficiency since they consider modesty a cherished virtue in their culture, the possibility that FL learning itself poses challenges to most Chinese FL learners seems to suffice.

### Relations Among Chinese High School Students’ Foreign Language Classroom Anxiety, Foreign Language Enjoyment, and Expectancy-Value Motivation

RQ2 considers the relation between Chinese high school students’ FLCA, FLE, and expectancy-value motivation. The participants’ responses showed that their FLCA was significantly negatively correlated while FLE was significantly positively correlated with expectancy beliefs, meaning that the participants who experienced lower FLCA and higher FLE when learning English were more likely to be driven by firmer expectancy beliefs, and vice versa. These results also resonate with some recent findings (e.g., Liu and Dong, 2021; Dong and Liu, 2022). But moreover, it was found that FLE was significantly positively correlated with all components of value motivation while FLCA was negatively correlated with intrinsic value and positively associated with cost value. It is likely that positive FL emotions help add to the value of the learning activity and open learners to more learning chances, while negative FL emotions expose learners to wakening their weaknesses, thus making them balk at learning tasks and debark from their FL learning interest and engagement. Alternatively, according to the broaden-and-build theory (Fredrickson and Joiner, 2018), positive emotions open the mind and nourish the growth of resources, while negative emotions such as anxiety or anger have opposite narrowing effects (Dewaele and Li, 2021).

As shown in Figures 2, 3, the participants whose FL classroom emotions differed also varied sharply with regard to expectancy-value appraisals, lending credence to the interaction of FL classroom emotions and expectancy-value motivation (Xu, 2017; Liu and Dong, 2021). Our study further revealed that as the participants’ FLCA level increased, their expectancy beliefs, intrinsic value, attainment value, and utility value decreased, while their cost value increased. In contrast, as their FLE increased, their expectancy beliefs, intrinsic value, attainment value, and utility value all increased, while their cost value first increased and then decreased. These results can be interpreted in two possible ways. First, it is understandable that since cost value mainly pertains to the negative aspects of doing a task that requires time, effort, and emotional investment (Eccles and Wigfield, 2020), the more FL learners feel anxious about completing a task, the more they need to wrestle with aspects even irrelevant to the task itself, which certainly consumes resources available for working memory (Carroll, 1999). Hence, not surprisingly, the participants’ perceived cost value increased with their anxious feelings. However, the increase of FLE may not necessarily translate into decreased cost value but only helps downsize it when FLE itself is at a high level. This means that only when FL learners’ FLE is high will they perceive FL learning as more cost-effective. Second, this finding may corroborate Dewaele and MacIntyre’s (2014) and Dewaele et al.’s (2019b) conclusion that FLE and FLCA are not opposite ends of the same dimension and are indeed influenced by differentiated factors, with FLE more associated with teacher-related variables while FLCA more linked to learners themselves. Our results support these ideas by further showing that the increase in FLE may significantly lift all components of expectancy-value motivation while the decrease of FLCA may only significantly increase expectancy beliefs and intrinsic value. Explanations for this can also be drawn from a few previous studies that weighed the effects of FLE on FL learning against those of FLCA and reported that the facilitative effects of FLE, both cross-sectional and longitudinal, were stronger than the debilitative effects of FLCA when they were examined simultaneously (e.g., Saito et al., 2018; Li and Li, 2022).

### Predictive Effects of Foreign Language Classroom Anxiety, Foreign Language Enjoyment, and Expectancy-Value Motivation on Self-Rated Foreign Language Proficiency

RQ3 addresses how the participants’ FLCA, FLE, and components of expectancy-value motivation predicted their self-rated FL proficiency. As Model 4 in Table 4 suggests, the participants’ self-rated FL proficiency was specifically predicted by a selection of expectancy-value motivation and classroom emotion components. Precisely, expectancy beliefs and intrinsic value are the motivational factors that predicted learners’ self-rated FL proficiency, concurring with Loh’s (2019) claim that competency beliefs and interests are two synchronous ideas for many young students. Expectancy beliefs are learners’ evaluations of how well they expect themselves to do in the future based on their previous experience (Wigfield and Eccles, 2000). They are more like cumulated and therefore stable judgments closely related to individuals’ self-rated ability. Another predictor, intrinsic value, is the inner gain learners can obtain from participating in an activity or completing a task (Wigfield and Eccles, 2000). According to the self-determination theory, language learners’ intrinsic motivation reflects their inherent inclination toward carrying out learning tasks (Alamer, 2021b), which in the meanwhile, is also indicative of the fun, pleasure, and excitement in performing the task *per se* (Noels et al., 2003; Alamer, 2021a). Naturally, those with high intrinsic value tend to be more motivated to learn, both psychologically and behaviorally. Likewise, when individuals do intrinsically valued tasks, they will also gain important consequences, most of which are quite positive (Deci and Ryan, 1985). Such a significant role of intrinsic motivation in learning performance has been well endorsed across gender, ethnicity, and institutional levels in educational settings (see Ryan and Deci, 2020). Apart from motivational factors, we found that private FLE and FLCA caused by fear of negative evaluation were two FL emotion predictors of self-rated FL proficiency. These two dimensions deal with the personal side of FL classroom emotions. Hence, they exhibited bearings with self-rated FL proficiency which is also a psychologically rooted factor. Furthermore, although private FLE significantly positively predicted while FLCA caused by fear of negative evaluation negatively predicted self-rated FL proficiency, the predictive power of the former outweighed that of the latter, confirming the findings of some previous studies (e.g., Saito et al., 2018; Li and Li, 2022). As Dewaele et al. (2019b) firmly put, “it is FLE rather than FLCA that is directly tied to the product of successful L2 learning in classroom settings.” Before concluding, it is important to note that our research did not replicate the “expectancy × value” interaction as reported in a few recent studies (e.g., Zhan et al., 2020; Dong and Liu, 2022). This might be attributed to the differences in dependent variables under varied investigations. For example, our study did not relate the expectancy-value motivation components directly to real FL test performance and FL learners’ behaviors, which were assumed to be more strongly impacted by expectancy and value beliefs as we previously reviewed. Yet this finding adds some fresh insights to and merits further elaboration for the specificity of the “expectancy × value” interaction (Wan, 2019; Zhan et al., 2020), an intriguing avenue awaiting more future research.

## Conclusion

The present study investigated the relations among FLCA, FLE, expectancy-value motivation, and their predictive effects on Chinese high school students’ self-rated FL proficiency. It was found that (1) Chinese high school students generally experienced a medium to a high level of FLCA and FLE in their FL learning, with the latter slightly higher than the former. Chinese high school students were overall more value-motivated than expectancy-motivated in FL learning, and most of them perceived their FL proficiency as unsatisfying; (2) Chinese high school students’ FLCA was significantly negatively correlated, while FLE was positively correlated with their expectancy beliefs. Whereas their FLE was significantly positively correlated with all components of value motivation, their FLCA and value components of motivation demonstrated a complex correlational pattern; and (3) Chinese high school students’ expectancy beliefs, intrinsic value, private FLE, and FLCA caused by fear of negative evaluation jointly predicted their self-rated FL proficiency.

As the present study highlights the significant positive predictive effects of learners’ language learning enjoyment and expectancy-value motivation on FL learning outcomes, these findings have pedagogical implications. First, it is necessary, if at all possible, that task value interventions are implemented and integrated with FL teaching and learning. Research in neighboring fields has already illustrated that task value interventions could promote learning interest and engagement (e.g., Hulleman et al., 2017). Language teachers can draw on the idea by purposefully raising their learners’ task value awareness. It is especially recommended that they encourage language learners to align subjective appraisals of FL learning activities/tasks to personal goals in such forms as short essay writing, dialog journals, or group sharing (Nagle, 2021). Second, language teachers may consider devising approaches to boost FL learners’ private FLE experience. To do so, aside from creating an enjoyable FL learning environment where FL learners feel safe and supported, teaching efforts should also be made to enhance FL learners’ positive feelings about their own progress in FL learning. Third, given the strong predictive effects of intrinsic value motivation on FL learning, cultivating FL learners’ intrinsic value gains particular urgency. A feasible way may be that more interest-arousing elements are incorporated into FL learners’ learning materials to gift learners not only with good grades but also with positive feelings. On the way to achieving this, there is the hope that we are making strides toward bolstering our language learners’ character growth and emotional wellbeing (MacIntyre et al., 2019).

Our findings should be considered in light of at least three limitations. First, since the research data were collected in a convenience sampling process from only one school, homogeneity in the region, participants’ age, teachers’ instructional style, and school effects are inevitable. Therefore, the relations between variables as evidenced by the present study may not be generalizable to a larger EFL or FL learner population. For this reason, future research is needed to verify our findings in a wider pool of participants from various contexts. Second, we only considered self-rated FL proficiency in the study because of the difficulty in organizing a standardized and reliable proficiency test. Our participants might be prone to underestimate their proficiency due to modesty. Further research can map out solutions to address this issue. Third, it is worth noting that the present study’s findings were based on mere cross-sectional data. Thus, we do not ascertain that they reflected causal relations among our measured variables. We look forward to more longitudinal research assessing the relations among FL emotions, learning motivation, and language proficiency springing up in the future. Notwithstanding these limitations, the present study serves to enrich the literature on how FL classroom emotions, expectancy-value motivation, and self-rated FL proficiency link and coexist in the process of FL learning.

## Data Availability Statement

The raw data supporting the conclusions of this article will be made available by the authors, without undue reservation.

## Author Contributions

LD conceived the idea, analyzed the data, and drafted the manuscript. ML provided critical feedback and helped to shape the research. FY reviewed and revised the manuscript. All authors contributed to the article and approved the submitted version.

## Conflict of Interest

The authors declare that the research was conducted in the absence of any commercial or financial relationships that could be construed as a potential conflict of interest.

## Publisher’s Note

All claims expressed in this article are solely those of the authors and do not necessarily represent those of their affiliated organizations, or those of the publisher, the editors and the reviewers. Any product that may be evaluated in this article, or claim that may be made by its manufacturer, is not guaranteed or endorsed by the publisher.
